# Motor and higher‐order functions topography of the human dentate nuclei identified with tractography and clustering methods

**DOI:** 10.1002/hbm.25551

**Published:** 2021-06-04

**Authors:** Fulvia Palesi, Matteo Ferrante, Marta Gaviraghi, Anastasia Misiti, Giovanni Savini, Alessandro Lascialfari, Egidio D'Angelo, Claudia A. M. Gandini Wheeler‐Kingshott

**Affiliations:** ^1^ Department of Brain and Behavioral Sciences University of Pavia Pavia; ^2^ Department of Physics University of Pavia Pavia Italy; ^3^ Department of Electrical, Computer, and Biomedical Engineering University of Pavia Pavia Italy; ^4^ Department of Neuroradiology IRCCS Humanitas Research Hospital Milan Italy; ^5^ Brain Connectivity Center IRCCS Mondino Foundation Pavia; ^6^ NMR Research Unit, Queen Square MS Centre, Department of Neuroinflammation UCL Queen Square Institute of Neurology London

**Keywords:** cerebellum, connectivity, dentate nuclei, diffusion imaging, microstructure, MRI, thalamus, tractography

## Abstract

Deep gray matter nuclei are the synaptic relays, responsible to route signals between specific brain areas. Dentate nuclei (DNs) represent the main output channel of the cerebellum and yet are often unexplored especially in humans. We developed a multimodal MRI approach to identify DNs topography on the basis of their connectivity as well as their microstructural features. Based on results, we defined DN parcellations deputed to motor and to higher‐order functions in humans in vivo. Whole‐brain probabilistic tractography was performed on 25 healthy subjects from the Human Connectome Project to infer DN parcellations based on their connectivity with either the cerebral or the cerebellar cortex, in turn. A third DN atlas was created inputting microstructural diffusion‐derived metrics in an unsupervised fuzzy c‐means classification algorithm. All analyses were performed in native space, with probability atlas maps generated in standard space. Cerebellar lobule‐specific connectivity identified one motor parcellation, accounting for about 30% of the DN volume, and two non‐motor parcellations, one cognitive and one sensory, which occupied the remaining volume. The other two approaches provided overlapping results in terms of geometrical distribution with those identified with cerebellar lobule‐specific connectivity, although with some differences in volumes. A gender effect was observed with respect to motor areas and higher‐order function representations. This is the first study that indicates that more than half of the DN volumes is involved in non‐motor functions and that connectivity‐based and microstructure‐based atlases provide complementary information. These results represent a step‐ahead for the interpretation of pathological conditions involving cerebro‐cerebellar circuits.

## Graphical abstract

We developed a multimodal MRI approach to identify DNs topography on the basis of their connectivity, in turn with either the cerebral or the cerebellar cortex, as well as their microstructural features, inputting them in an unsupervised fuzzy c‐means classification algorithm. The three approaches provided quite overlapping results in terms of geometrical distribution and, for the first time, indicate that more than half of the DN volumes is involved in non‐motor higher‐order functions.

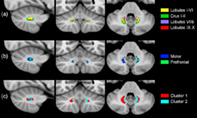

## 1 INTRODUCTION

The brain is the principal organ of the nervous system and is composed of several different neuron types that are structurally and functionally well‐organized to create specialized tissues (Voogd & Ruigrok, 2012). Glial cells and neurons are spatially organized in layers which mainly compose the outer part of the brain, the cortex. Axons and dendrites, instead, are organized in coherent bundles belonging to the inner part of the brain, the white matter. Other gray matter structures, called deep gray matter nuclei, are identifiable within the white matter and are extremely important because they are synaptic relays, meaning that they represent areas where neurons make synapses achieving information transfer and integration. Hence, these regions are responsible to route signals, transported along the axons, to and from specific areas of the brain.

Cerebellar nuclei represent important synaptic areas, which are often unexplored in in vivo imaging studies of the human brain. Indeed, they are crucial hubs for cerebro‐cerebellar and spino‐cerebellar communication (Hámori, 1977; Voogd & Ruigrok, 2012). The biggest cerebellar nuclei are the two dentate nuclei (DN), which are the farthest from the cerebellar midline and one at either hemisphere. Most of the efferent cerebellar connections towards the cerebral cortex synapse in the DN then convey in the superior cerebellar peduncle and pass through the contralateral red nucleus to end in the thalamus (Voogd & Ruigrok, 2012). Commonly, the DNs are known to be involved in sensorimotor processes but recent functional imaging investigations have revealed that they play a role also in non‐motor functions (Alahmadi et al., 2017; Bharti et al., 2020; Habas, Guillevin, & Abanou, 2011; Zhang et al., 2015). This finding is in line with the recent understanding that the cerebellum is connected to cognitive and associative cortical areas, as supported by either tract‐tracing techniques (Kelly & Strick, 2003; Middleton & Strick, 1994; Schmahmann & Caplan, 2006; Schmahmann & Pandya, 1995; Strick, Dum, & Fiez, 2009a) or diffusion MRI tractography studies of the cerebro‐cerebellar loop (Kim, Im, Kim, & Park, 2019; Palesi et al., 2015, 2016, 2017).

To characterize the DN topography, histochemical and viral tract‐tracing studies have demonstrated that in animals (e.g., rats, cats, and apes) the DNs present a topographical organization based on their cerebellar cortical connectivity (Matano, 2001; Obadiah, 2015). Two distinct zones can be identified: one rostro‐dorsal and one ventro‐caudal, associated to motor and non‐motor functions, respectively. This result represents a step forward in our understanding of the role of cerebellar nuclei on brain function; nevertheless, given that the cerebellar cortical organization in humans is much more complex than in animals, one has to question what is the DN topography in this case.

To our knowledge, only a study using noninvasive methods has been performed in humans in‐vivo to assess DN topography (Steele et al., 2017). Here, sub‐millimeter diffusion MR images of the cerebellum of six volunteers were acquired on a 7 T scanner. Probabilistic tractography was used to parcellate DNs in humans on the basis of the cerebellar region that was mostly connected: motor rostro‐dorsal and non‐motor ventro‐caudal areas were identified. This was a promising result because supported the fact that the DNs present a topography reflecting different functional properties, but the main limit was that this study focused only on DN connectivity with the cerebellum instead of considering the whole‐brain. Furthermore, tractography suffers of some intrinsic limitations and a multimodal approach could provide a mean of validating results. Worth considering that in the last decade machine learning has been widely applied to MRI analysis either to support clinical diagnosis or to improve images accuracy and has been proven to be capable to segment specific brain structures, including recently the DNs, as a whole, with a higher accuracy compared to other automatic methods (Gaviraghi et al., 2021).

In this study, therefore, we aimed to use a whole‐brain MRI‐based multimodal approach to reconstruct and investigate the topography of the DNs. Constrained spherical deconvolution tractography (Tournier, Calamante, & Connelly, 2007) was performed to reconstruct connections between DNs and both cerebral and cerebellar cortices, in turns, deriving two independent connectivity‐based atlases of the DNs. Furthermore, a clustering approach, based on a fuzzy c‐means algorithm, was developed to provide insights about DN topography based on diffusion MRI microstructural properties. The final goal of this study was to compare the atlases obtained with these three approaches and propose a coherent sub‐parcellation of the DNs in humans in‐vivo, with particular focus on assessing the DNs percentage deputed to motor and to non‐motor functions. The resulting DN atlas would be of great interest also for improving structural connectivity investigations in humans; indeed, these cerebellar structures are currently not included in atlases used for anatomically constrained tractography (Smith, Tournier, Calamante, & Connelly, 2012). Given their role in the cerebro‐cerebellar connectivity, we believe they should be included in future tractography studies as a synaptic gray matter structure, similarly to what is done with the thalamus. Needless to say that a more correct anatomical connectome of the brain would also impact studies that are investigating brain dynamics (Palesi et al., 2020).

## 2 MATERIALS AND METHODS

### 2.1 Subjects

Minimal pre‐processed images of 25 healthy subjects (16 females and nine males) were downloaded from those acquired for the Human Connectome Project (HCP; http://db.humanconnectome.org; Van Essen et al., 2013), with age comprised between 26 and 35 years.

### 2.2 MRI acquisition

The MRI protocol was setup on a Siemens MAGNETOM Skyra 3 T scanner, adapted with high field gradients, using a 32‐channel receive head coil. Diffusion weighted images (DWI) were acquired with a multi‐shell spin‐echo EPI sequence with these parameters: TR = 5,520 ms, TE = 89.5 ms, flip angle = 78°, FOV = 210 × 180 mm^2^, 111 axial slices, 1.25 mm isotropic voxel, three *b* values of 1000, 2000, and 3000 s/mm^2^, 18 images with null *b* value (b0 images), and 90 isotropically distributed diffusion directions per *b* values. A co‐registered 3DT1‐weighted image was also downloaded.

### 2.3 MRI preprocessing and DN masks definition

Downloaded DWI data were already preprocessed for noise removal and motion correction in native space. Diffusion tensor (DT) and DT‐derived metrics, such as Fractional Anisotropy (FA), Mean Diffusivity (MD), Axial and Radial Diffusivity (AD, RD), were calculated with FSL (FMRIB Software Library, https://fsl.fmrib.ox.ac.uk/fsl/fslwiki; Jenkinson, Beckmann, Behrens, Woolrich, & Smith, 2012). Mean, Axial, and Radial Kurtosis (MK, AK, RK) maps were extracted with DESIGNER (https://github.com/NYU-DiffusionMRI/DESIGNER; Ades‐Aron et al., 2018).

DN masks were extracted from b0 images using a convolutional neural network (CNN) as described by Gaviraghi et al. (2021). This automatic, machine learning based method was proven to be able to extract more accurate DNs masks than other methods using standard atlases and templates.

3DT1‐weigthed images were segmented in native space in gray matter (GM), deep GM nuclei, white matter (WM), and cerebrospinal fluid using the 5ttgen algorithm (MRtrix3, https://www.mrtrix.org/; Tournier et al., 2019). To improve the reliability of the segmentation and tractography, DN masks were added to the deep GM mask per each subject. Then, WM‐GM interface was also calculated.

Those 3DT1‐weighted images were warped to the standard MNI152 template (Fonov et al., 2011) by concatenating an affine (12 dof, FLIRT, FSL; Jenkinson, Bannister, Brady, & Smith, 2002) and a nonlinear (FNIRT, FSL; Andersson, Jenkinson, & Smith, 2010) transformation.

The transformation matrix “subject‐to‐MNI space” was then inverted (MNI‐to‐subject) to be applied to specific atlases to warp them back to the subject space for the subsequent DN parcellation work (Sections 2.5 and 2.6).

### 2.4 Whole‐brain tractography

The fiber orientation density function was evaluated in native space separately for each tissue using the multi‐tissue multi‐shell approach (Dhollander, Raffelt, & Connelly, 2016; Jeurissen, Tournier, Dhollander, Connelly, & Sijbers, 2014). Then, a whole‐brain probabilistic anatomically constrained tractography (Smith et al., 2012) was performed with 30 million streamlines seeded dynamically from the WM‐GM interface and cropped to the WM mask. Other parameters were: step of 0.0625 mm (corresponding to half of voxel‐size), maximum length of 250 mm, and maximum angle of 45° between two subsequent points of the same streamline, to avoid unrealistic torsions and backpropagations.

### 2.5 Cerebellar lobule‐specific connectivity to the DN

**FIGURE 1:**
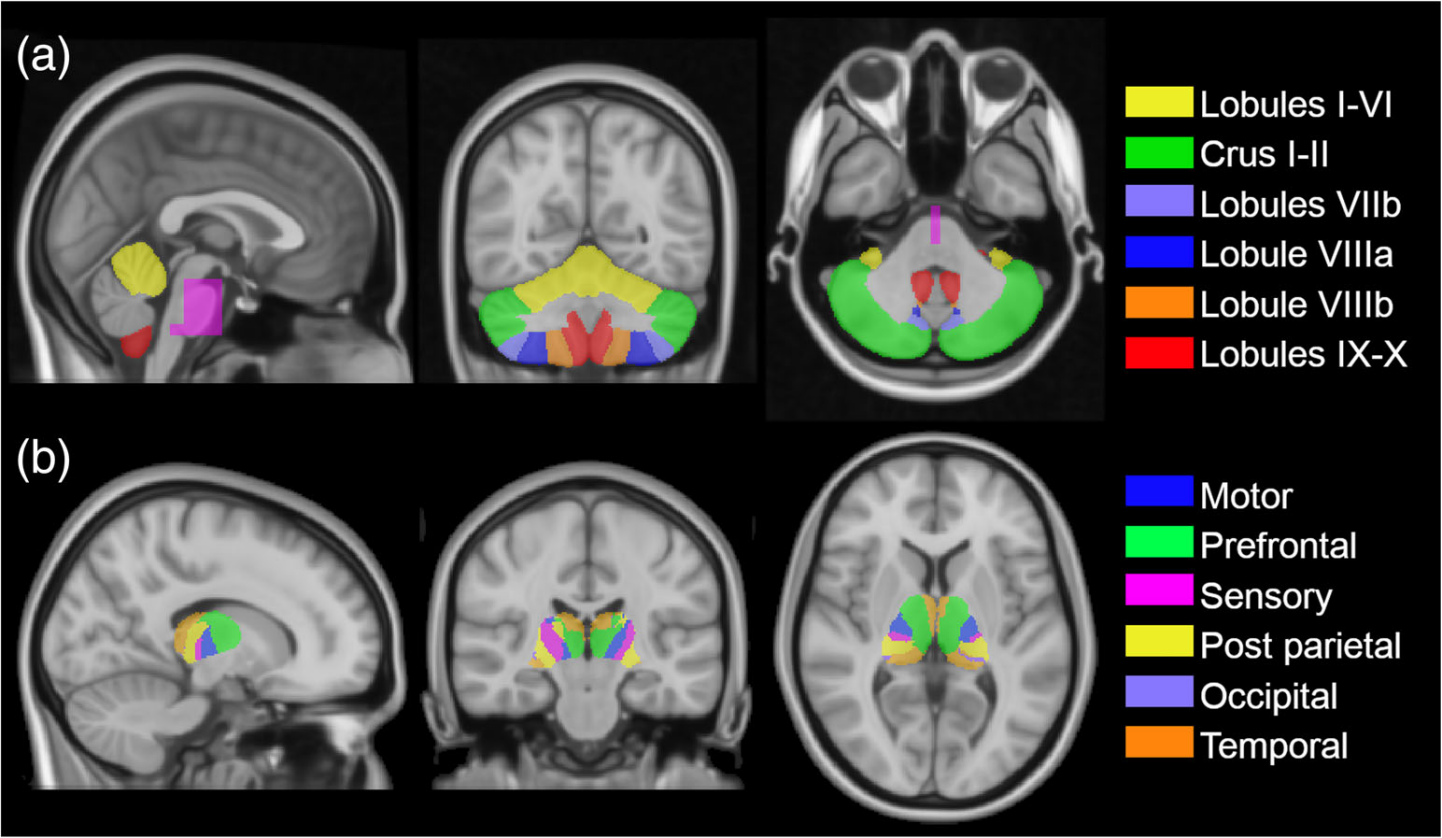
Atlases and NOT‐ROI used to identify cerebellar and thalamic connectivity with the DNs. All maps were in standard MNI152 space (radiological view) and were superimposed on the standard T1 weighted image. Panel A shows the sagittal NOT‐ROI (fuchsia), used to avoid false positives in tractography, and the cerebellar parcellations: lobules I–VI (yellow), Crus I–II (green), lobule VIIb (violet), lobule VIIIa (blue), lobule VIIIb (orange), and lobules IX–X (red). Panel B shows the thalami parcellation based on the atlas described by Behrens et al. (2003, 2003): motor (blue), prefrontal (green), sensory (fucshia), posterior parietal (yellow), occipital (violet), and temporal (orange)

To define masks of different cerebellar lobules, the “Spatially unbiased atlas template of the cerebellum and brainstem” (SUIT; Diedrichsen, Balsters, Flavell, Cussans, & Ramnani, 2009) was warped to the subject space applying the MNI‐to‐subject transformation. We then merged some of the parcellations based on their known function, creating a final atlas with six parcellations per hemisphere: lobules I–VI (motor area), Crus I–II (cognitive area), lobule VII, lobule VIIIa, lobule VIIIb, and lobules IX–X (visuo‐spatial functions; Figure 1). The vermis was discarded because it is directly connected with the fastigial nucleus instead of the DNs (Voogd & Ruigrok, 2012).

From the whole‐brain tractography, we selected tracts terminating in each of the identified cerebellar parcellation and connected to their ipsilateral DN. Based on anatomy, no structural internal connection between the two cerebellar hemispheres exists (Pollok et al., 2006), hence an excluding region of interest (NOT‐ROI) was placed aligned with the medial plane of the cerebellum to avoid spurious inter‐hemispheric streamlines (Figure 1).

This step provided a lobule‐specific connectivity map, defining a set of streamlines per cerebellar lobule departing from each of the six parcellations of the cerebellar cortex, which was used to generate the first subject‐specific topography map of each DN, on the basis of their structural connectivity with the ipsilateral cerebellar cortex.

### 2.6 Subthalamic‐specific connectivity to the DN

The second subject‐specific DN topography map was defined based on the DN connectivity to the cerebral hemisphere, rather than cerebellar cortex. Most of the cerebellar output connections pass through the DN and synapse into the contralateral thalamus before reaching the cerebral cortex. Because our tractography was anatomically constrained, only monosynaptic streamlines were reconstructed. Thus, we used the contralateral thalamus as target to study the connections between the DNs and the contralateral cerebral cortex. Thalamic parcellation in native space was defined by applying the MNI‐to‐subject transformation to the atlas defined by Behrens, Johansen‐Berg, et al. (2003); Behrens, Woolrich, et al. (2003), whose classification was made on the basis of connectivity between the thalamus and the cerebral cortex. Since primary motor and pre‐motor regions were merged for the cerebellar cortex parcellation, we did the same for the thalamus where six ROIs were identified: motor, prefrontal, sensory, posterior parietal, occipital, and temporal (Figure 1).

Subthalamic‐specific tracts were selected from the whole‐brain tractography using the DN and the contralateral subthalamic areas as target ROIs.

### 2.7 Connectivity‐based topography atlases of the DN

For each subject, the cerebellar lobule‐specific and subthalamic‐specific topography atlases of DNs were created following several steps, as described below. The same steps performed to reconstruct the cerebellar lobule‐specific atlas were repeated, as reported in square brackets, to reconstruct the subthalamic‐specific atlas.

**FIGURE 2:**
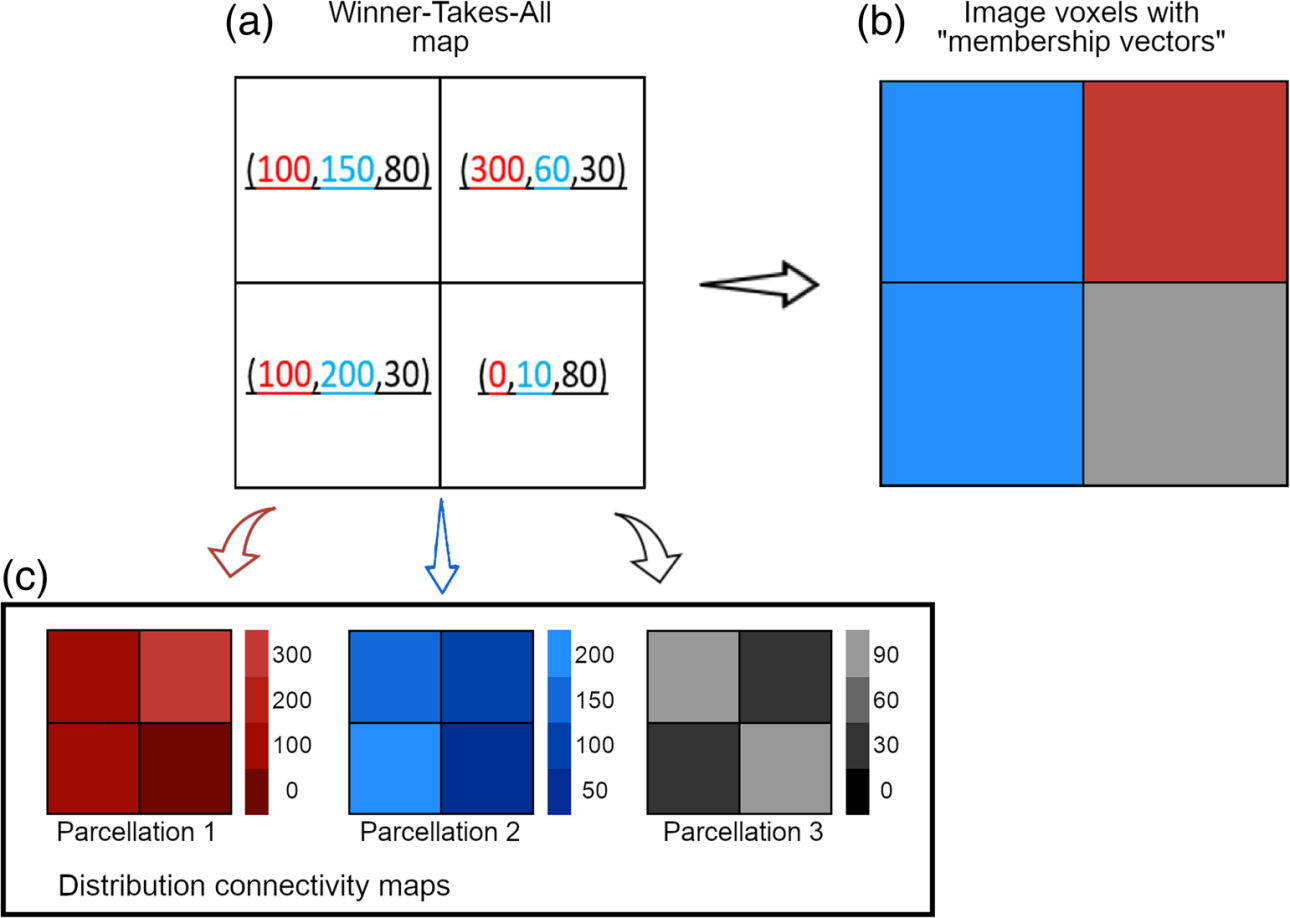
Simplified graphical representation for the creation of the DNs topography atlases. As an example, the case of a mask with four voxels (a) associated to three different parcellations (b) is considered. Panel A reports the four voxels with their “membership vectors”: red numbers indicate the number of streamlines connecting the first parcellation to each voxel, blue numbers indicates those connecting the second parcellation to each voxel, and black numbers those connecting the third parcellation to each voxel. Panel B shows the final topography atlas obtained with the winner‐takes‐all algorithm. Panel C shows the distribution connectivity maps for each specific parcellation, which are used to support the result in B

First, a track‐density image (TDI), namely an image whose intensity is proportional to the number of streamlines passing through each voxel, was calculated for each of the parcellation‐specific tract (Calamante, Tournier, Jackson, & Connelly, 2010). Then, TDI maps were masked with the ipsilateral (contralateral) DN and for each voxel of the mask a “membership vector” was defined by the connection strength of each cerebellar lobule (thalamus‐parcellation) to each DN voxel (Figure 2a). The “membership vector” had as many components as the number of parcellations, and the number associated to each component corresponded to the number of streamlines per parcellation that ended in that specific voxel. This vector was used to assign to each voxel of the DN the parcellation most likely to be connected to it, following a winner‐takes‐all rule (Figure 2b). The association was supported by the comparison between the resulting DN parcellations and the distribution connectivity maps, computed from the “membership vector” component specific to each parcellation (Figure 2c).

After membership assignment, the number of streamlines of tracts connecting the DNs to each parcellation and volume of each parcellation were calculated for each subject.

### 2.8 Microstructure‐based topography atlas of the DN

An unsupervised clustering approach was performed on diffusion‐derived metrics to generate a topography map of the DNs where parcellations are obtained on the basis of voxel‐wise local microstructural features. All diffusion‐derived metrics were masked with the DN masks to obtain a dataset of microstructural features: each data point represented a voxel of the DN, with a vector of features per voxel generated from maps of different microstructural properties. For each subject, each feature was normalized to its maximum and the interquartile range method (Gholizadeh et al., 2019) was applied to replace outliers with median values.

The resulting dataset was used as input to a fuzzy c‐means (FCM) clustering algorithm in MATLAB routine (Fuzzy Logic toolbox—Data Clustering, https://it.mathworks.com/help/fuzzy/fcm.html; Bezdek, 1981). Differently from the k‐means algorithm, which is a hard clustering method, the decision boundary of the fuzzy c‐means is softer and the output for each data point is the probability of belonging to each cluster. After several tests, qualitatively optimal results were obtained using three clusters as the number of parcellations, while the fuzziness parameter was set as default (*m* = 2; Campello & Hruschka, 2006). To create a topography map of the DNs based on the clustering of microstructural features, each DN voxel was assigned to the most probable cluster selected with a maximum likelihood algorithm.

Volumes of each cluster were calculated for each subject.

### 2.9 Inter‐subject topography atlases of the DNs

DN topography maps of all subjects were warped to a common space to achieve inter‐subject DN topography based on structural connectivity and microstructural features. Since DNs are small structures within the large brain volume, the SUIT ROI‐driven normalization algorithm was used (www.diedrichsenlab.org/imaging/suit_function.htm#norm_dentate), where the DNs masks have been inputted to drive the warping to the common SUIT space for the cerebellum, forcing a good overlap between deep cerebellar nuclei of different subjects in common space. Normalized DN masks of all subjects were visually inspected to confirm alignment across subjects.

For each subject, a numeric label was associated to each parcellation of the cerebellar lobule‐specific and subthalamic‐specific topography map. Then, their inter‐subject topography atlases were created assigning, to each voxel of the DNs, the mode of the numerical label across all subjects, as a standard approach when averaging categorical values.

To create the inter‐subject topography map based on microstructural features, we first defined a regulatory function (bottom‐up hierarchic classifier) that tooks as input the atlas found with the fuzzy c‐means algorithm, to provide up to three nonordered clusters for each subject. Given that DNs were separated into three parcellations only in a subset of subjects, a posteriori we reduced the number of clusters to two in order to improve reproducibility. In this way, a medial and a lateral cluster were identified and labeled in the same order for all subjects. Then, the mode of the values across subjects was calculated to generate the inter‐subject microstructure‐based atlas.

### 2.10 Atlas comparison

To further investigate the comparison between the different topography atlases of DNs found with different approaches, we computed the DICE similarity coefficient (DSC) between all the parcellations of the three atlases in standard space. The DSC between two parcellations was computed doubling the intersection volume over the sum of the two volumes (Prados et al., 2015)

$$\mathrm{DSC}\left(A,B\right)=2\frac{\left|A\cap B\right|}{\left|A\right|+\left|B\right|}$$

### 2.11 Statistical analysis of DN properties

Parcellations of the DNs were quantitatively assessed in terms of their microstructural features to determine differences or similarities between left and right side and parcellations; normative values were also determined for future studies.

Statistical tests were performed using SPSS software version 21 (IBM, Armonk, New York).

All features previously calculated (number of streamlines and volumes as well as diffusion‐derived metrics) were tested for normality (Shapiro–Wilk test). Most of them were nonnormally distributed, thus a nonparametric Wilcoxon test for paired variables was applied to assess statistically significant differences between the left and right side. Furthermore, mean FA, MD, and MK were calculated for each connectivity‐based DN atlas and a nonparametric Friedman test was applied to assess whether significant differences between microstructural features exist between different parcellations. Lastly, as an exploratory investigation all these features were compared with a non‐parametric Mann–Whitney test (*p* < .05) between females and males, in order to assess whether gender‐related differences exist.

## 3 RESULTS

Connectivity‐based and microstructure‐based topography atlases of the DNs were successful in demonstrating parcellations consistent with predicted expectations from animal work. Motor and cognitive/associative areas were identified based on connectivity with cerebellar and thalamus parcellations. Underlying microstructure features also identified distinct regions with main differences between the lateral and medial parcellations of DNs. Left and right DNs were symmetrical in terms of their quantitative properties.

### 3.1 Cerebellar lobule‐specific topography atlas of the DN

**FIGURE 3:**
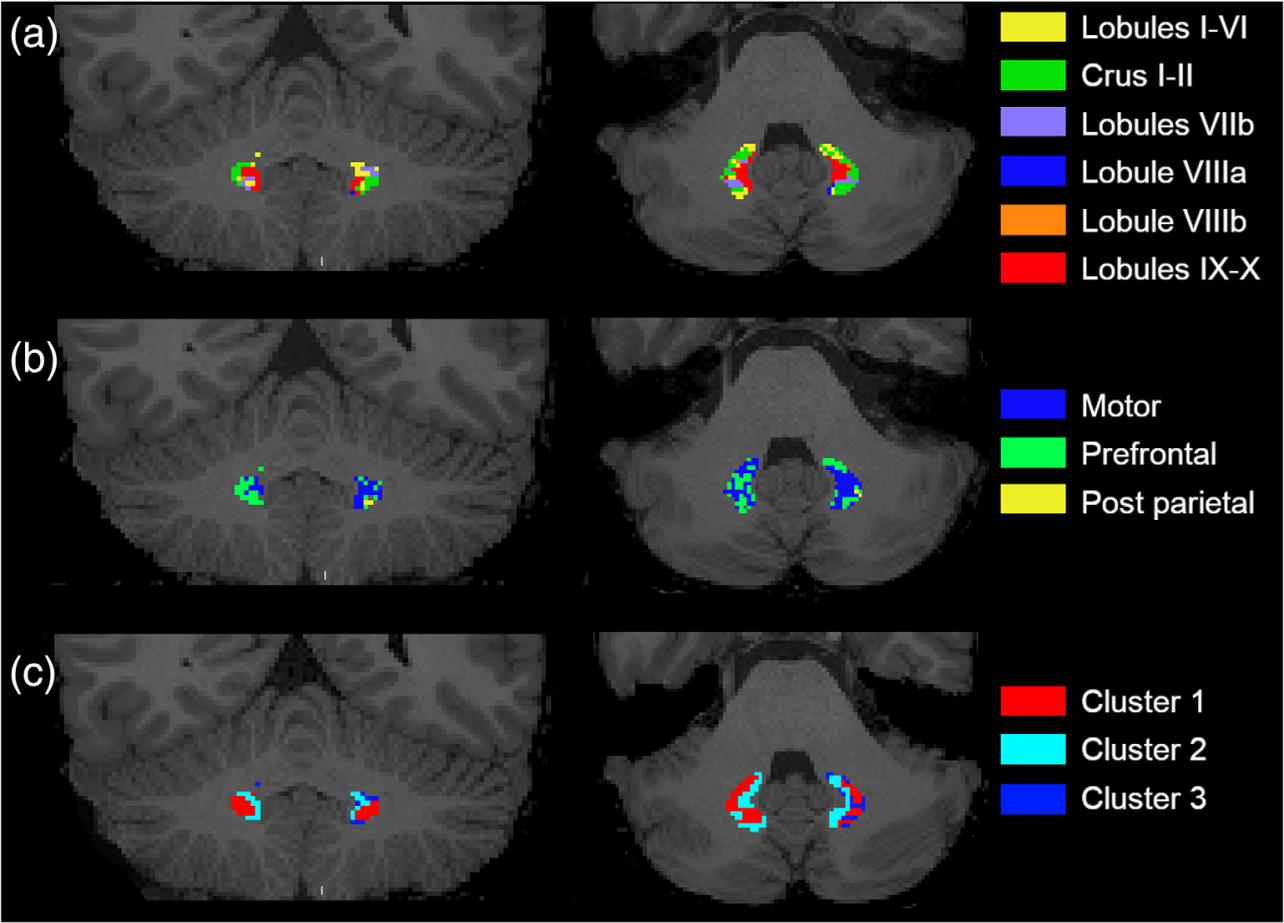
(a, b) Connectivity‐based and (c) microstructure‐based topography atlases of the DNs in a randomly chosen subject (radiological view). Color code is the same as the other figures. Panel A shows the cerebellar lobule‐specific DN atlas, Panel B shows the subthalamic‐specific DN atlas, while Panel C shows the microstructure‐based DN atlas

An example of cerebellar lobule‐specific topography atlas of the DNs in a random chosen subject is shown in Figure 3a.

**FIGURE 4:**
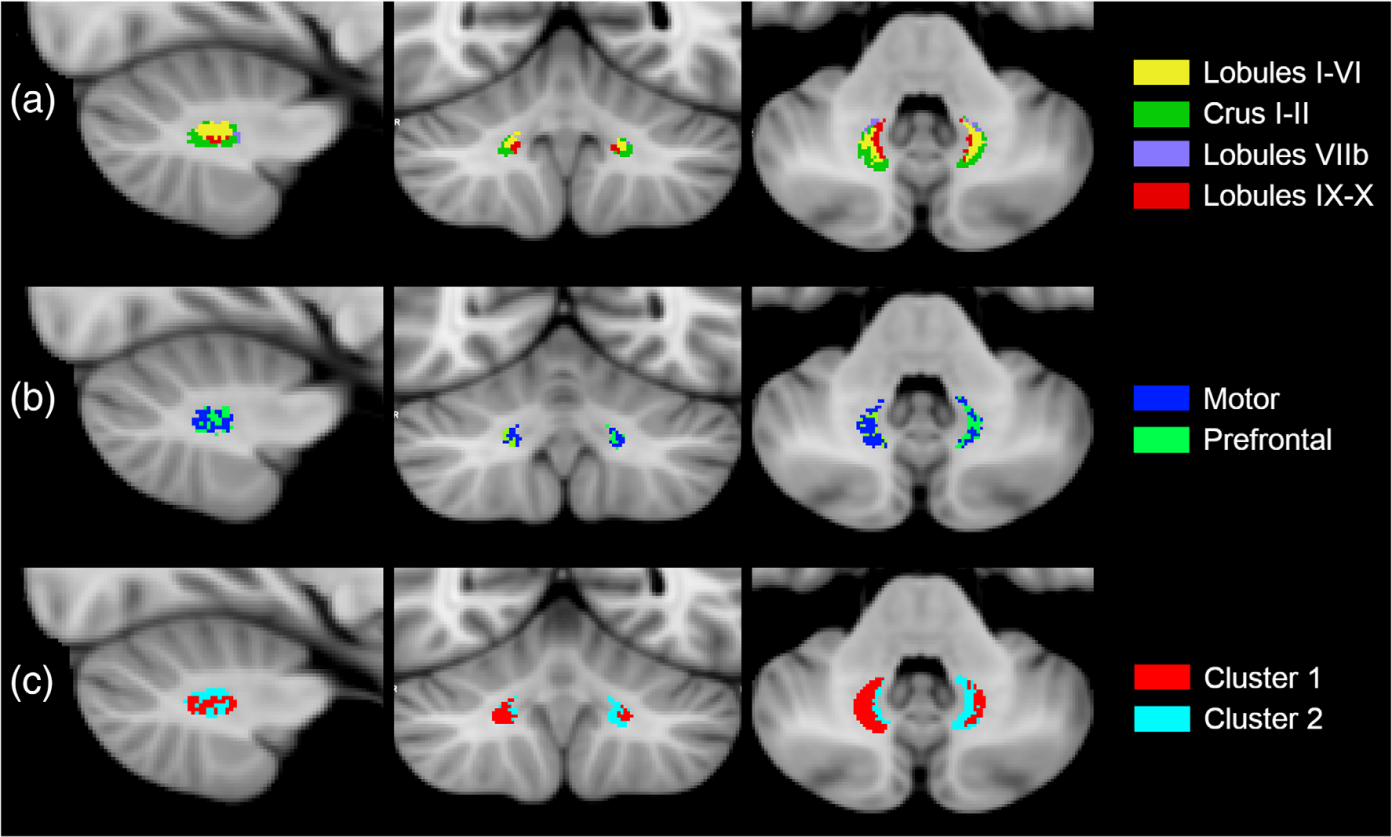
Inter‐subject topography atlases of the DNs in the MNI152 space (radiological view). Panel A shows cerebellar lobule‐specific parcellations of the left and right DNs: the medial portion is associated with lobules IX–X (red), more laterally with lobules I–VI (yellow), the most lateral portion is associated with Crus I–II (green), the ventral part is associated with lobule VII (violet). Panel B shows subthalamic‐specific parcellations of the left and right DNs: the medial part is associated with prefrontal cortex (green) while the lateral part is associated with motor cortex (blue). Panel C shows left and right DN parcellations based on microstructural metrics: the biggest cluster (cluster 1) occupies the lateral part of the DNs (red) while the other cluster (cluster 2) occupies the medial part of DNs (light blue)

Group atlas in SUIT cerebellar space showed an overall good agreement between subjects as shown by Figure 4a. Although we started from six parcellations, only three were characterized by more than 10,000 streamlines connecting them to specific cerebellar lobules and with a parcellation volume >15% of the overall DN volume; these three parcellations were those connecting the DN to lobules I‐VI, Crus I‐II and lobules IX‐X of the cerebellar cortex. Lobule VIIb showed a few consistent overlapping voxels across subjects, while lobules VIIIa and VIIIb did not identify a consistent area as their connectivity produced smaller clusters or even isolated voxels (Figure 4a). The main results in terms of statistical descriptive features, averaged across all subjects, are summarized in Table 1 (streamlines) and Table 2 (volumes).

**TABLE 1:** Number of streamlines between cerebellar and thalamic parcellations and DNs

Atlas	Parcellation	Left DN		Right DN		*p* value
		Mean ± *SD*	Range	Mean ± *SD*	Range	
Cerebellar lobule‐specific (×10^3^)	Lobule I–VI	18.9 ± 7.9	12.5–24.2	18.4 ± 7.7	13.8–22.2	.968
	Crus I–II	38.0 ± 12.4	29.0–45.0	49.6 ± 13.6	38.1–54.8	**<.001**
	Lobule VII	9.2 ± 4.6	6.4–10.4	8.0 ± 4.2	5.5–10.6	.128
	Lobule VIIIa	2.7 ± 2.2	1.1–4.3	2.3 ± 2.1	0.9–2.8	.288
	Lobule VIIIb	2.0 ± 2.9	0.4–1.9	1.6 ± 2.4	0.3–1.6	.427
	Lobule IX–X	11.8 ± 8.1	6.6–13.4	13.4 ± 6.1	9.2–16.8	.109
Subthalamic‐specific (×10^3^)	Motor	0.33 ± 0.31	0.10–0.49	0.40 ± 0.34	0.19–0.47	**.019**
	Prefrontal	0.39 ± 0.31	0.14–0.60	0.41 ± 0.31	0.17–0.54	.476
	Sensory	0.05 ± 0.04	0.03–0.07	0.04 ± 0.05	0.01–0.05	.002
	Posterior parietal	0.06 ± 0.06	0.01–0.09	0.08 ± 0.07	0.03–0.11	.014
	Occipital	0.001 ± 0.002	0–0.001	0.003 ± 0.003	0–0.006	.001
	Temporal	0.03 ± 0.02	0.01–0.04	0.02 ± 0.02	0.01–0.03	.333

*Note*: Across‐subject mean number of streamlines that reached the left and right DNs. The number of streamlines are expressed in ×10^3^. Range is defined as 25th to 75th percentiles. Bold *p* values (Wilcoxon test) are statistically significant and considered meaningful.

**TABLE 2:** Volume of DN parcellations according to connectivity‐ and microstructure‐based topography atlases

Atlas	Parcellation	Left DN		Right DN		*p* value
		Mean ± *SD*	Range	Mean ± *SD*	Range	
Cerebellar lobule‐specific	Lobule I–VI	28.9 ± 8.6	23.1–34.1	27.4 ± 11.4	18.9–37.9	.737
	Crus I–II	39.5 ± 14.3	30.4–50.3	41.7 ± 10.7	32.3–48.0	.737
	Lobule VII	8.0 ± 7.0	1.9–11.3	8.7 ± 8.1	1.5–14.1	.968
	Lobule VIIIa	3.0 ± 2.9	0.9–5.0	2.3 ± 2.4	0.4–3.6	.150
	Lobule VIIIb	4.2 ± 4.5	0.8–6.5	2.2 ± 3.2	0.1–3.4	.020
	Lobule IX–X	16.4 ± 8.4	10.7–22.8	17.8 ± 8.1	12.8–21.2	.545
Subthalamic‐specific	Motor	45.9 ± 19.8	34.5–61.2	53.4 ± 15.8	39.9–67.8	.069
	Prefrontal	44.8 ± 19.4	29.4–53.7	37.2 ± 16.0	25.5–47.3	**.030**
	Sensory	3.8 ± 4.1	1.0–5.3	1.7 ± 1.9	0.4–1.8	.005
	Posterior parietal	4.7 ± 5.7	0.8–5.3	7.0 ± 7.2	2.6–8.6	.026
	Occipital	0.1 ± 0.1	0.0–0.0	0.1 ± 0.1	0.0–0.0	.138
	Temporal	0.7 ± 0.8	0.0–1.1	0.6 ± 0.1	0.0–0.7	.485
Microstructure‐based	Cluster 1 (lateral)	56.0 ± 24.6	45.0–69.8	53.7 ± 29.4	35.2–81.1	.716
	Cluster 2 (medial)	44.0 ± 24.7	30.2–55.0	46.3 ± 29.4	18.9–64.8	.716

*Note*: Across‐subject mean volumes in left and right DNs. Values are expressed in percentage of the overall DN volume. Range is defined as 25th to 75th percentiles. Bold *p* value (Wilcoxon test) is statistically significant.

In detail, for each DN, the biggest parcellation covered the most lateral portion, accounted for about 40,000 streamlines and 40% of the DN volume and was associated with the Crus I–II. The second relevant parcellation was located rostro‐medially to the biggest one, accounted for about 20,000 streamlines and 30% of the DN volume and was associated to lobules I–VI. The third parcellation occupied, mainly caudally, the most medial area of the DN and was linked to lobules IX–X, accounting for around 10,000 streamlines and 15% of the DN volume.

The number of streamlines connecting the right Crus I–II and its ipsilateral DN was statistically significantly higher (*p* < .001) than the left one while no differences were identified between left and right DNs volumes for any parcellation.

### 3.2 Subthalamic‐specific topography atlas of the DN

An example of subthalamic‐specific topography atlas of the DNs in a random chosen subject is shown in Figure 3b.

Inter‐subject subthalamic‐specific topography atlas of the DNs showed an overall good agreement between subjects as shown by Figure 4b. Although we started from six parcellations, only two were characterized by more than 300 streamlines connecting them to specific subthalamic areas and with a parcellation volume >40% of the overall DN volume; these two parcellations were those that indirectly connect the DN to motor and prefrontal cortex. The other subthalamic areas did not identify a consistent area as their connectivity produced isolated voxels. The main results, averaged across all subjects, are summarized in Table 1 (streamlines) and Table 2 (volumes).

In detail, for each DN, the biggest parcellation accounted for about 46% of the DN volume and was associated with projections to the ventrolateral and ventroanterior part of the thalamus and, indirectly, with the cerebral motor cortex. The second relevant parcellation counted for about 45% of the DN volume and was associated with projections to the mediodorsal part of the thalamus and, indirectly, with the prefrontal cortex.

The right DN showed significantly higher number of streamlines connected with the contralateral motor area (*p* < .019) and smaller volume associated with the contralateral prefrontal cortex (*p* = .030).

### 3.3 Microstructure‐based atlas of the DN

An example of microstructure‐based atlas of the DNs in a random chosen subject is shown in Figure 3c.

Inter‐subject microstructure‐based atlases of the DNs were obtained using as input voxel‐wise feature vectors of FA, MD, AD, RD, MK, AK, RK metrics computed from DWI data. Results show that this method divided the DNs in two parcellations, despite asking the algorithm to find up to 3.

The main results, averaged across all subjects, are summarized in Table 2 and shown in Figure 4c.

For each DN, the biggest parcellation accounted for about 55% of the DN volume and was located laterally, whereas the second parcellation counted for about 45% of the DN volume and was located medially.

No statistically significant differences were identified between left and right DN volumes.

### 3.4 Atlas comparison

**FIGURE 5:**
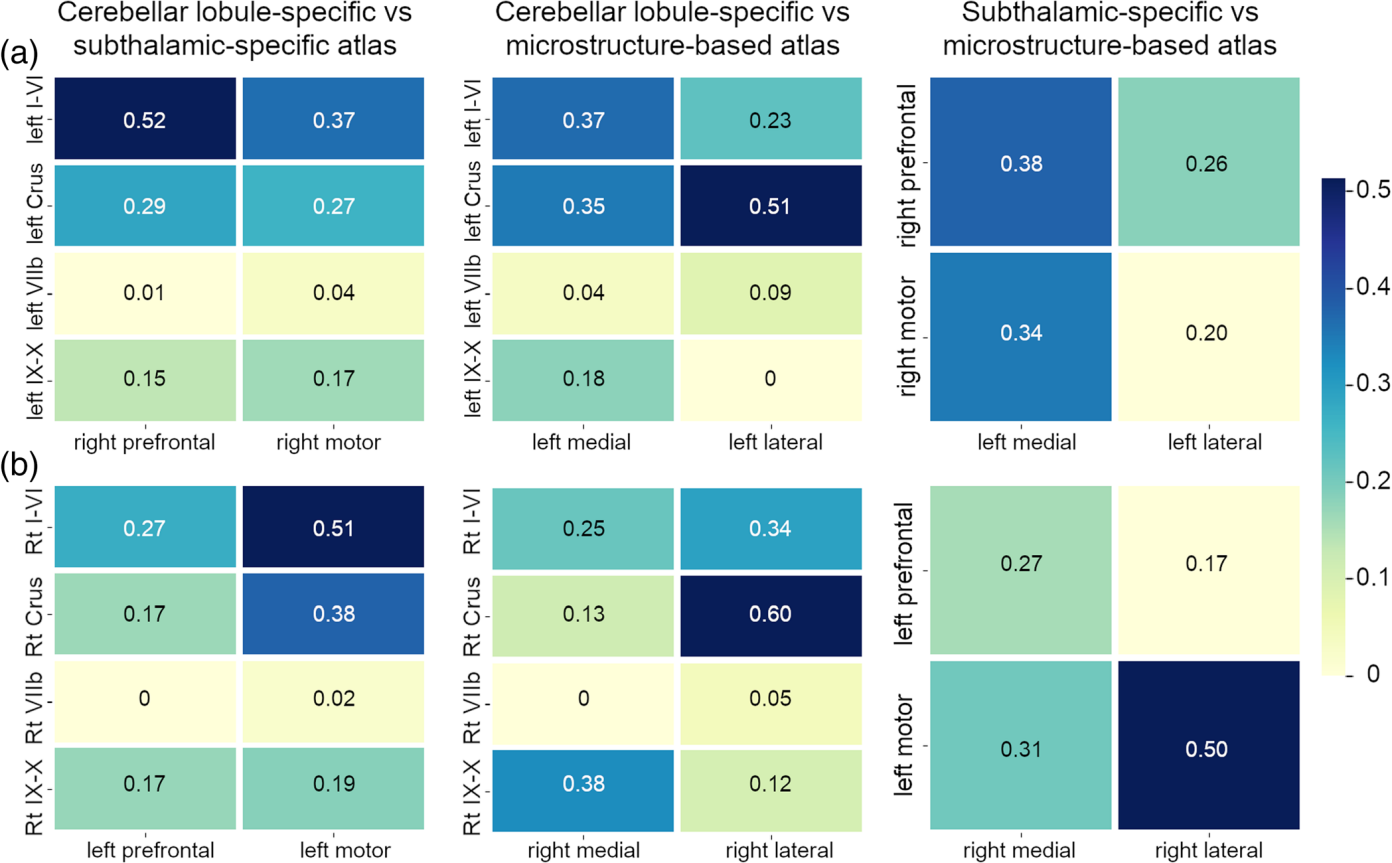
Inter‐subject DICE similarity coefficient (DSC) representation. Panel A shows DSC values for left DN parcellations, while panel B shows DSC values for right DN parcellations. From left to right, are shown DSC values between cerebellar lobule‐specific and subthalamic‐specific parcellations (left), cerebellar lobule‐specific and microstructure‐based parcellations (center), subthalamic‐specific and microstructure‐based parcellations (right)

Inter‐subject DSC between each parcellation of different topography DN atlases are reported in Figure 5. Panel A reports DSC for the left DN, while panel B reports those for the right DN. From left to right, each row shows DSC between cerebellar lobule‐specific and subthalamic‐specific parcellations, then between cerebellar lobule‐specific and microstructure‐based parcellations, and between subthalamic‐specific and microstructure‐based parcellations.

Overall, DSC ranged between 0 and 1. The highest value (0.60) was found between right Crus I‐II and the lateral parcellation when cerebellar lobule‐specific and microstructure‐based topography atlases were compared, while the smallest values (<0.1) were found comparing lobule VIIb with the parcellations of the other two approaches. The highest DSC values were found when parcellations representing either the connectivity with lobules I‐VI or Crus I–II were overlapped to the others.

### 3.5 Diffusion‐derived metrics in cerebellar lobule‐specific DN parcellations

Given the outcome of the subthalamic‐specific connectivity, we decided to assess microstructural features only for the DN parcellations obtained with cerebellar lobule‐specific connectivity.

**FIGURE 6:**
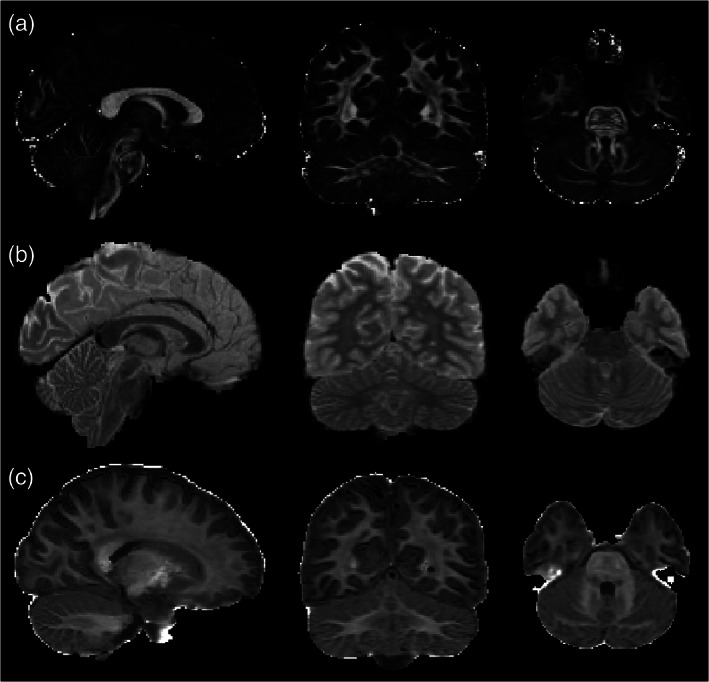
Diffusion‐derived metrics maps (FA, MD, and MK) in a randomly chosen subject (radiological view). Panel A shows FA map, Panel B shows MD map, while Panel C shows MK map

Across subjects mean values of diffusion‐derived metrics (FA, MD, and MK) in cerebellar lobule‐specific parcellations are reported in Table 3. We choose those parameters because they were the less correlated diffusion‐derived metrics in the computed set of FA, MD, AD, RD, MK, AK, RK. Those three metrics are shown for a randomly chosen subject in Figure 6.

**TABLE 3:** Microstructure features of the DN connectivity‐based parcellations

	Metric	Lobule I–VI	Crus I–II	Lobule VII	Lobule VIIIa	Lobule VIIIb	Lobule IX‐X	*p* value
Left DN	FA	0.276 ± 0.048	0.292 ± 0.038	0.277 ± 0.052	0.318 ± 0.103	0.320 ± 0.102	0.276 ± 0.053	.210
	MD (×10^−3^ mm^2^/s)	0.805 ± 0.171	0.753 ± 0.164	0.756 ± 0.164	0.758 ± 0.172	0.767 ± 0.189	0.820 ± 0.177	**<.001**
	MK	1.155 ± 0.056	1.213 ± 0.078	1.213 ± 0.097	1.215 ± 0.100	1.195 ± 0.087	1.165 ± 0.059	**<.001**
Right DN	FA	0.294 ± 0.045	0.308 ± 0.026	0.315 ± 0.076	0.328 ± 0.083	0.362 ± 0.088	0.274 ± 0.029	**.013**
	MD (×10^−3^ mm^2^/s)	0.818 ± 0.175	0.76 ± 0.164	0.734 ± 0.163	0.738 ± 0.172	0.742 ± 0.178	0.827 ± 0.179	**<.001**
	MK	1.150 ± 0.072	1.221 ± 0.093	1.227 ± 0.110	1.202 ± 0.127	1.194 ± 0.097	1.147 ± 0.068	**<.001**

*Note*: Across‐subject mean diffusion‐derived metrics (fractional anisotropy [FA], mean diffusivity [MD], mean kurtosis [MK]) in lobule‐based parcellations. Bold *p* values (Friedman test) are statistically significantly different.

All metrics demonstrated statistically significant differences between cerebellar lobule‐specific parcellations of the DNs. FA was significantly different only between parcellations of the right DN (*p* = .013), while MD and MK values were significantly different between both left and right DNs parcellations (*p* < .001).

### 3.6 Gender‐related effect on connectivity and microstructure features

Gender‐related differences were found mainly in the left DN, as reported in Table 4.

**TABLE 4:** Gender effect on connectivity and microstructure features of the DN parcellations

Parameter	Females	Males	*p* value
	Mean ± *SD*	Mean ± *SD*	
Streamlines LeftDN‐CrusI‐II (×10^3^)	33.0 ± 10.5	46.9 ± 11.0	.008
Streamlines LeftDN‐Thal‐Motor (×10^3^)	0.42 ± 0.35	0.18 ± 0.15	.043
Volume LeftDN‐Lobule VIIIb	0.06 ± 0.05	0.01 ± 0.01	.002
Volume LeftDN‐Cluster 1 (lateral)	0.49 ± 0.27	0.69 ± 0.14	.012
Volume LeftDN‐Cluster 2 (medial)	0.51 ± 0.27	0.31 ± 0.14	.012
FA LeftDN‐Lobule VIIIa	0.279 ± 0.072	0.388 ± 0.116	.023
MD LeftDN‐Lobule VIIIa (×10^−3^ mm^2^/s)	0.813 ± 0.072	0.662 ± 0.251	.007
MK LeftDN‐Lobule VIIIa	1.175 ± 0.083	1.286 ± 0.090	.008
FA RightDN‐CrusI‐II	0.300 ± 0.026	0.322 ± 0.020	.023
MD RightDN‐Lobule VIIIa (×10^−3^ mm^2^/s)	0.792 ± 0.060	0.648 ± 0.253	.020

*Note*: Across‐subject mean features (i.e., number of streamlines, volumes and diffusion‐derived metrics) for the specified parcellation. Only features showing significant *p* values (Mann–Whitney test) between females and males are reported.

The number of the streamlines between the left DN and the ipsilateral Crus I‐II was significantly higher in males with respect to females, while the number of those connecting the left DN and the contralateral motor cortex was significantly higher in females with respect to males.

Volume of the two parcellations defined with the microstructure‐based atlas was strongly dependent on gender for the left DN: the lateral cluster was larger in males while the medial cluster was larger in females.

Diffusion‐derived metrics in cerebellar lobule‐specific parcellations demonstrated statistically significant differences in the left and right Lobule VIIIa. FA and MK were lower in females while MD was higher in females with respect to males.

## 4 DISCUSSION

The present study represents one of the few investigations focused on the topography of the DN based on its structural connectivity to cerebellar lobules or subthalamic nuclei as well as on its microstructural properties, and assessing whether such DNs parcellations compartmentalize motor and non‐motor functions. The main observation is that using three different parcellation approaches it was possible to identify two or more DN parcellations, in humans in‐vivo, on the basis of structural connectivity and microstructural features. Our findings also suggest that, globally, in both left and right DNs, the non‐motor representation is predominant on the motor one. It is to note that, although the predominance of the higher‐order functions representation is maintained, the motor component is predominant in females than males.

Previous studies on animals (Matano, 2001; Obadiah, 2015) have demonstrated a dichotomy in the DN role by identifying one rostro‐dorsal and one ventro‐caudal zone related to motor and non‐motor functions, respectively. A similar parcellation was also identified in humans by Steele et al. (2017). Here, we reconstructed two different structural atlases of human DNs based on their connectivity either with cerebellar or cerebral cortex, by performing a state‐of‐the‐art probabilistic tractography. A third atlas, which identified two different clusters, was obtained by inputting diffusion‐derived metrics in a unsupervised clustering algorithm reflecting underlying microstructural properties.

Cerebellar lobule‐specific connectivity provided a DNs topography atlas with three main clusters whose location was reproducible between subjects. The biggest parcellation was located laterally and dorso‐caudally in both DNs and was the one connected with the Crus I‐II, which represent the most important (Strick, Dum, & Fiez, 2009b; Voogd & Ruigrok, 2012) cognitive areas of the cerebellum. This parcellation involved more than 40,000 streamlines and occupied about 40% of the total volume of each DN. It is worth noting that this finding might support the one reported by Palesi et al. (2015), which asserted that almost 50% of the Crus I‐II cortex was involved in the cerebello‐thalamo‐cortical tract. Furthermore, our results suggest that this parcellation may be affected by gender, as males showed a higher number of streamlines connecting the cerebellar cortex to the left DN compared to females. This may suggest that, although the higher‐order functions parcellation is the largest in all subjects, the relative percentage between motor and higher‐order functions areas depends on gender. The medial‐caudal parcellation of both DNs occupied about 15% of the DNs volume and involved connections with lobules IX–X that represent non‐motor areas, supporting mainly sensory functions. Then, the rostral area between the previous ones represented the motor related parcellation, namely the one connected with lobules I‐VI, and accounted for about 30% of DNs volume. The remaining volume was associated to mixed connections from lobules VIIb and lobules VIII. These results indicate that the most represented parcellation is related to high‐level cognitive processes, the second is related to motor functions, followed by a third parcellation mainly deputed to sensory and associative functions. Globally, this implicates that more than half of the DNs volume is involved in non‐motor functions. These finding are in agreement and further improve those reported by Steele et al. (2017), which investigated only connectivity between DNs and lobules I–VI and Crus I–II.

It is interesting to compare these results with those obtained with the unsupervised fuzzy c‐means algorithm, which identified two separate clusters, one medial and one lateral, on the basis of microstructural features of each DNs voxel. These clusters are reproducible across subjects and overlap quite well with those identified with cerebellar lobule‐specific connectivity despite some differences in volumes. Indeed, the lateral cluster includes the cognitive representation of the DN, while the medial one comprises the sensory representation. It is interesting that the motor representation, as identified by connectivity‐based approaches, in the microstructure‐based atlas is either included in the lateral cluster, for the right DN, or in the medial one, for the left DN. However, the volume of this lateral cluster is bigger than the one related to Crus I‐II connectivity meaning that a mixture of cognitive and motor representations always coexists within it. This hypothesis seems to be confirmed by the fact that, in the left DN, females showed a bigger medial cluster, which contains both the sensory and motor components, while males showed a bigger lateral cluster, which mainly supports higher‐order functions. Furthermore, mean values of diffusion‐derived microstructure metrics in cerebellar lobule‐specific parcellations (Table 3) reveal the presence of a unique trend for both DNs where motor and sensory (lobules IX–X) parcellations have more similar microstructural features than Crus I–II. Nevertheless, relative errors are quite large and mean values of features overlap, which could explain the uncertainty of the inclusion of the motor representation in either the lateral/medial cluster for right/left DN. Higher resolution data, in terms of both geometrical and diffusion characterization, could help understanding the source of this left/right difference.

Moreover, the topography atlas based on cerebral and subthalamic‐specific connectivity represented the most challenging approach that provided the worst reproducible DN atlas. This method was able to define two main parcellations connected with the mediodorsal, ventral anterior and ventro‐lateral thalamic nuclei, which are directly connected with the prefrontal cortex (the first two nuclei) and the motor/premotor area (the last nucleus). Again, this atlas identified two separate components related to non‐motor and motor functions but the location of these clusters was not well defined across different subjects. There were streamlines starting from adjacent voxels that end in different areas of thalamus, making the generation of the subthalamic‐specific parcellation very sensible to this effect. As shown in Figures 3b and 4b, those topography atlases are asymmetric between left and right DNs. Different confounders might have an influence on this result, which could be attributed to the fact that the biophysical nature of the connections between each DN and its ipsilateral cerebellar cortex or each DN and its contralateral thalamus is substantially different, with methodological repercussions. In the former case, the connections are short, nondecussating and involving fairly big areas, the cerebellar lobules. In the latter case, instead, we are considering challenging long tracts that involve small areas (the DN and subthalamic nuclei) and contain axonal fibers that pass through a decussation point where dense fibers cross each other. Furthermore, some axonal bundles may branch within the thalamus, resulting in one single axon connecting to more than one subthalamic nucleus. Recent study has highlighted the effect of different fiber orientation distribution (FOD) in representing the underlying fiber geometry (Canales‐Rodríguez et al., 2019). Some variability of our results may be introduced by the fact that we used a well‐established tractography package (MRtrix) and its default FOD (iFOD2), which may have intrinsic limitations in representing some specific geometrical configurations. Furthermore, it is to remember that tractography has intrinsic limitations always affecting the reconstruction of both unexplored as well as known connections, with the main drawbacks being the inability to discern directionality of signal propagation along the reconstructed pathways, the reconstruction of false positive tracts or the inability to reconstruct true tracts (false negative), and the difficulty of reconstructing crossing and polysynaptic tracts (Maier‐Hein et al., 2017; Nath et al., 2020; Schilling et al., 2019).

Aside of how to define the DN atlas, interesting considerations come from microstructural properties of DN parcellations, which demonstrates a substantial left–right symmetry of DNs atlases, and confirmed differences between the two connectivity‐based topography approaches. In detail, the cerebellar lobule‐specific connectivity shows that the cognitive representation is bigger in volume than the motor one with a significant increased number of streamlines connecting he cerebellar cortex with the right DN. Instead, the subthalamic‐specific connectivity method identified similar volumes for the motor and the prefrontal representations in the left DN, while the right DN was characterized by a significant smaller volume for the prefrontal representation and a significant increased number of streamlines connecting the motor area. These results may be due to the technical challenges of the subthalamic‐specific connectivity; on the other hand, there could be a physiological selectivity of motor and premotor functions that are attributed to each DN and are causing this asymmetry in distribution. Future studies should try to untangle such ambiguity that this study cannot resolve.

Lastly, it is not surprising that connectivity‐based and microstructure‐based approaches provide similar but not identical atlases because they intrinsically contain different but complementary information. In particular, cerebellar lobule‐specific connectivity was able to distinguish between motor and non‐motor representations, while the second demonstrated that motor related parcellation was characterized by microstructural features similar to those of the sensory parcellation, leading them to be indistinguishable. Further studies should better investigate this last open question in order to provide stronger and more specific evidence of microstructural organization underlying brain functioning. In this context, emerging microstructural models, such as SANDI (Palombo et al., 2020), might be useful to characterize neurite specific proprieties both in white and gray matter structures.

It is worth noting that present parcellations coming from diffusion MRI analyses might grow their significance by integrating them with other techniques able to quantify specific biological features, such as myelin or iron content. This procedure would allow to merge structural and molecular proprieties information providing a step ahead in the interpretation of specific mechanisms underlying pathologies that are known to affect DNs and cerebellar circuitry, such as cerebellar ataxia and autistic spectrum disorders. Moreover, it is becoming apparent that the cerebellum plays a key role in neurodegenerative pathologies and other neurological diseases, for example, Alzheimer's disease (Castellazzi et al., 2014; Palesi et al., 2018) and epilepsy (Rolandi et al., 2021; Streng & Krook‐Magnuson, 2020). For example, it is known that the cerebellum and basal ganglia are connected through a polysynaptic connection and that the cerebellum plays a compensative role on the basal ganglia in Parkinson's disease (Simioni, Dagher, & Fellows, 2016; Yu, Sternad, Corcos, & Vaillancourt, 2007). A full characterization of the DNs in healthy and pathological conditions will contribute to the investigation of mechanisms of damage and repair in a number of different pathologies.

It is clear that all these findings will further improve our anatomical and physiological understanding of the cerebro‐cerebellar loops, also providing a more comprehensive structural characterization of brain circuitry. Indeed, the DN atlas provided by this work could be embedded into one of the tissue classes (e.g., the deep gray matter class) used in anatomically constraint tractography framework or in other tractography algorithms using anatomical priors (Schiavi et al., 2020), if data is acquired with a voxel resolution comparable to that of the HCP data. Moreover, using the DN atlas to define multiple nodes within each DN might be useful to identify specific DN connections with cerebellar lobules deputed to specific functions. This knowledge will also support the development of new tools and brain signal models for understanding and predicting brain functioning (Friston et al., 2019; Sanz Leon et al., 2013). In the last years, indeed, growing investigations have developed complex and multiscale approaches with the common goal of employing brain structural proprieties, such as diffusion MRI, to predict brain functional dynamics at single subject's level in both normal and pathological conditions (Palesi et al., 2020; Schirner, Mcintosh, Jirsa, & Deco, 2018; Zimmermann et al., 2018).

## 5 CONCLUSION

For the first time, the topography of the human DNs was defined on the basis of their own connectivity both with the cerebellar cortex and the thalamus, which is the main relay towards the cerebral cortex, as well as by exploiting their microstructural features. Our findings demonstrated that more than half of the DNs volume is involved in non‐motor functions, and two distinct regions were consistently identified: one deputed to high‐level cognitive processes and one identified with sensory functions. It is to note that these components were affected, although only in the left DN, by gender: females demonstrated a more developed motor component while males showed a larger area supporting higher‐order functions. Although connectivity‐based and microstructural‐based parcellations were in agreement, it is to note that the resulting atlases contained different and complementary information. We speculate that these results should be incorporated into current knowledge on the structural and functional significance of the cerebro‐cerebellar loops in humans, also providing novel insights that could be applied for interpretating pathological conditions affecting these circuits.

## CONFLICT OF INTEREST

The authors declare no conflict of interest.

## Data Availability

The raw data supporting the conclusions of this article will be made available without undue reservation, by contacting the corresponding author.

## References

[hbm25551-bib-0001] Ades‐Aron, B., Veraart, J., Kochunov, P., McGuire, S., Sherman, P., Kellner, E., … Fieremans, E. (2018). Evaluation of the accuracy and precision of the diffusion parameter EStImation with Gibbs and NoisE removal pipeline. NeuroImage, 183, 532–543. 30077743 10.1016/j.neuroimage.2018.07.066 PMC6371781

[hbm25551-bib-0002] Alahmadi, A. A. S., Pardini, M., Samson, R. S., Friston, K. J., Toosy, A. T., D'Angelo, E., & Gandini Wheeler‐Kingshott, C. A. M. (2017). Cerebellar lobules and dentate nuclei mirror cortical force‐related‐BOLD responses: Beyond all (linear) expectations. Human Brain Mapping, 38, 2566–2579. 28240422 10.1002/hbm.23541 PMC5413835

[hbm25551-bib-0003] Andersson JLR, Jenkinson M, Smith S (2010): Non‐linear registration, aka spatial normalization (FMRIB technical report TR07JA2).

[hbm25551-bib-0004] Behrens, T. E. J., Johansen‐Berg, H., Woolrich, M. W., Smith, S. M., Wheeler‐Kingshott, C. A. M., Boulby, P. A., … Matthews, P. M. (2003). Non‐invasive mapping of connections between human thalamus and cortex using diffusion imaging. Nature Neuroscience, 6, 750–757. 12808459 10.1038/nn1075

[hbm25551-bib-0005] Behrens, T. E. J., Woolrich, M. W., Jenkinson, M., Johansen‐Berg, H., Nunes, R. G., Clare, S., … Smith, S. M. (2003). Characterization and propagation of uncertainty in diffusion‐weighted MR imaging. Magnetic Resonance in Medicine, 50, 1077–1088. 14587019 10.1002/mrm.10609

[hbm25551-bib-0006] Bezdek, J. (1981). Pattern recognition with fuzzy objective function algorithms. New York: Plenum Press.

[hbm25551-bib-0007] Bharti, K., Khan, M., Beaulieu, C., Graham, S. J., Briemberg, H., Frayne, R., … Kalra, S. (2020). Involvement of the dentate nucleus in the pathophysiology of amyotrophic lateral sclerosis: A multi‐center and multi‐modal neuroimaging study. NeuroImage Clinical, 28, 102385. 32871387 10.1016/j.nicl.2020.102385 PMC7476068

[hbm25551-bib-0008] Calamante, F., Tournier, J. D., Jackson, G. D., & Connelly, A. (2010). Track‐density imaging (TDI): Super‐resolution white matter imaging using whole‐brain track‐density mapping. NeuroImage, 53, 1233–1243. 20643215 10.1016/j.neuroimage.2010.07.024

[hbm25551-bib-0009] Campello, R. J. G. B., & Hruschka, E. R. (2006). A fuzzy extension of the silhouette width criterion for cluster analysis. Fuzzy Sets and Systems, 157, 2858–2875.

[hbm25551-bib-0010] Canales‐Rodríguez, E. J., Legarreta, J. H., Pizzolato, M., Rensonnet, G., Girard, G., Patino, J. R., … Daducci, A. (2019). Sparse wars: A survey and comparative study of spherical deconvolution algorithms for diffusion MRI. NeuroImage, 184, 140–160. 30193974 10.1016/j.neuroimage.2018.08.071

[hbm25551-bib-0011] Castellazzi, G., Palesi, F., Casali, S., Vitali, P., Sinforiani, E., Wheeler‐Kingshott, C. A. M., & D'Angelo, E. (2014). A comprehensive assessment of resting state networks: Bidirectional modification of functional integrity in cerebro‐cerebellar networks in dementia. Frontiers in Neuroscience, 8, 1–18. 25126054 10.3389/fnins.2014.00223 PMC4115623

[hbm25551-bib-0012] Dhollander T, Raffelt D, Connelly A (2016): Unsupervised 3‐tissue responsefunction estimation from single‐shell or multi‐shell diffusion MR datawithout a co‐registered T1 image. In: ISMRM Work Break Barriers Diffus MRI p. 5.

[hbm25551-bib-0013] Diedrichsen, J., Balsters, J. H., Flavell, J., Cussans, E., & Ramnani, N. (2009). A probabilistic MR atlas of the human cerebellum. NeuroImage, 46, 39–46. 19457380 10.1016/j.neuroimage.2009.01.045

[hbm25551-bib-0014] Fonov, V., Evans, A. C., Botteron, K., Almli, C. R., McKinstry, R. C., & Collins, D. L. (2011). Unbiased average age‐appropriate atlases for pediatric studies. NeuroImage, 54, 313–327. 20656036 10.1016/j.neuroimage.2010.07.033 PMC2962759

[hbm25551-bib-0015] Friston, K. J., Preller, K. H., Mathys, C., Cagnan, H., Heinzle, J., Razi, A., & Zeidman, P. (2019). Dynamic causal modelling revisited. NeuroImage, 199, 730–744. 28219774 10.1016/j.neuroimage.2017.02.045 PMC6693530

[hbm25551-bib-0016] Gaviraghi, M., Savini, G., Castellazzi, G., Palesi, F., Rolandi, N., Sacco, S., … Gandini Wheeler‐Kingshott, C. A. M. (2021). Automatic segmentation of dentate nuclei for microstructure assessment: Example of application to temporal lobe epilepsy patients & Computational Diffusion MRI., Mathematics and Visualization, Cham, Switzerland: Springer.

[hbm25551-bib-0017] Gholizadeh, N., Fuangrod, T., Greer, P. B., Lau, P., Ramadan, S., & Simpson, J. (2019). An inter‐centre statistical scale standardisation for quantitatively evaluating prostate tissue on T2‐weighted MRI. Australasian Physical & Engineering Sciences in Medicine, 42, 137–147. 30637607 10.1007/s13246-019-00720-1

[hbm25551-bib-0018] Habas, C., Guillevin, R., & Abanou, A. (2011). Functional connectivity of the superior human temporal sulcus in the brain resting state at 3T. Neuroradiology, 53, 129–140. 20924756 10.1007/s00234-010-0775-5

[hbm25551-bib-0019] Chan‐Palay, V. (1977). The cerebellar dentate nucleus. Cerebellar dentate nucleus. Berlin, Germany: Springer.

[hbm25551-bib-0020] Jenkinson, M., Bannister, P., Brady, M., & Smith, S. (2002). Improved optimization for the robust and accurate linear registration and motion correction of brain images. NeuroImage, 17, 825–841. 12377157 10.1016/s1053-8119(02)91132-8

[hbm25551-bib-0021] Jenkinson, M., Beckmann, C. F., Behrens, T. E. J., Woolrich, M. W., & Smith, S. M. (2012). FSL. NeuroImage, 62, 782–790. 21979382 10.1016/j.neuroimage.2011.09.015

[hbm25551-bib-0022] Jeurissen, B., Tournier, J. D., Dhollander, T., Connelly, A., & Sijbers, J. (2014). Multi‐tissue constrained spherical deconvolution for improved analysis of multi‐shell diffusion MRI data. NeuroImage, 103, 411–426. 25109526 10.1016/j.neuroimage.2014.07.061

[hbm25551-bib-0023] Kelly, R. M., & Strick, P. L. (2003). Cerebellar loops with motor cortex and prefrontal cortex of a nonhuman primate. The Journal of Neuroscience, 23, 8432–8444. 12968006 10.1523/JNEUROSCI.23-23-08432.2003 PMC6740694

[hbm25551-bib-0024] Kim, Y., Im, S., Kim, S. H., & Park, G. Y. (2019). Laterality of cerebellar afferent and efferent pathways in a healthy right‐handed population: A diffusion tensor imaging study. Journal of Neuroscience Research, 97, 582–596. 30582195 10.1002/jnr.24378

[hbm25551-bib-0025] Maier‐Hein, K. H., Neher, P. F., Houde, J. C., Côté, M. A., Garyfallidis, E., Zhong, J., … Descoteaux, M. (2017). The challenge of mapping the human connectome based on diffusion tractography. Nature Communications, 8, 1–13. 10.1038/s41467-017-01285-x PMC5677006 29116093

[hbm25551-bib-0026] Matano, S. (2001). Brief communication: Proportions of the ventral half of the cerebellar dentate nucleus in humans and great apes. The American Journal of Physical Anthropology, 114, 163–165. 11169906 10.1002/1096-8644(200102)114:2<163::AID-AJPA1016>3.0.CO;2-F

[hbm25551-bib-0027] Middleton, F. a., & Strick, P. L. (1994). Anatomical evidence for cerebellar and basal ganglia involvement in higher cognition function. Science (80−), 266, 458–461. 10.1126/science.7939688 7939688

[hbm25551-bib-0028] Nath, V., Schilling, K. G., Parvathaneni, P., Hainline, A. E., Huo, Y., Blaber, J. A., … Landman, B. A. (2020). Tractography reproducibility challenge with empirical data (TraCED): The 2017 ISMRM diffusion study group challenge. Journal of Magnetic Resonance Imaging, 51, 234–249. 31179595 10.1002/jmri.26794 PMC6900461

[hbm25551-bib-0029] Obadiah, B. (2015). Topography of deep cerebellar nuclei of the African Giant pouched rat (*Cricetomys gambianus*). International Journal of Brain and Cognitive Sciences, 2015, 28–32.

[hbm25551-bib-0030] Palesi, F., Tournier, J.‐D., Calamante, F., Muhlert, N., Castellazzi, G., Chard, D., … Wheeler‐Kingshott, C. A. M. (2015). Contralateral cerebello‐thalamo‐cortical pathways with prominent involvement of associative areas in humans in vivo. Brain Structure & Function, 220, 3369–3384. 25134682 10.1007/s00429-014-0861-2 PMC4575696

[hbm25551-bib-0031] Palesi, F., Tournier, J.‐D., Calamante, F., Muhlert, N., Castellazzi, G., Chard, D., … Wheeler‐Kingshott, C. A. M. (2016). Reconstructing contralateral fiber tracts: Methodological aspects of cerebello‐thalamo‐cortical pathway reconstruction. Functional Neurology, 31, 229–238. 28072383 10.11138/FNeur/2016.31.4.229 PMC5231885

[hbm25551-bib-0032] Palesi, F., de Rinaldis, A., Castellazzi, G., Calamante, F., Muhlert, N., Chard, D., … Gandini Wheeler‐Kingshott, C. A. M. (2017). Contralateral cortico‐ponto‐cerebellar pathways reconstruction in humans in vivo: Implications for reciprocal cerebro‐cerebellar structural connectivity in motor and non‐motor areas. Scientific Reports, 7, 12841. 28993670 10.1038/s41598-017-13079-8 PMC5634467

[hbm25551-bib-0033] Palesi, F., de Rinaldis, A., Vitali, P., Castellazzi, G., Casiraghi, L., Germani, G., … Micieli, G. (2018). Specific patterns of white matter alterations help distinguishing Alzheimer's and vascular dementia. Frontiers in Neuroscience, 12, 274. 29922120 10.3389/fnins.2018.00274 PMC5996902

[hbm25551-bib-0034] Palesi, F., Lorenzi, R. M., Casellato, C., Ritter, P., Jirsa, V., Wheeler‐kingshott, C. A. M. G., & Angelo, E. D. (2020). The importance of cerebellar connectivity on simulated brain dynamics. Frontiers in Cellular Neuroscience, 14, 1–11. 32848628 10.3389/fncel.2020.00240 PMC7411185

[hbm25551-bib-0035] Palombo, M., Ianus, A., Guerreri, M., Nunes, D., Alexander, D. C., Shemesh, N., & Zhang, H. (2020). SANDI: A compartment‐based model for non‐invasive apparent soma and neurite imaging by diffusion MRI. NeuroImage, 215, 116835. 32289460 10.1016/j.neuroimage.2020.116835 PMC8543044

[hbm25551-bib-0036] Pollok, B., Butz, M., Gross, J., Südmeyer, M., Timmermann, L., & Schnitzler, A. (2006). Coupling between cerebellar hemispheres: Behavioural, anatomic, and functional data. Cerebellum, 5, 212–219. 16997753 10.1080/14734220600621294

[hbm25551-bib-0037] Prados, F., Cardoso, M. J., Leung, K. K., Cash, D. M., Modat, M., Fox, N. C., … Ourselin, S. (2015). Measuring brain atrophy with a generalized formulation of the boundary shift integral. Neurobiology of Aging, 36, S81–S90. 25264346 10.1016/j.neurobiolaging.2014.04.035 PMC4288791

[hbm25551-bib-0038] Rolandi, N., Palesi, F., Padelli, F., Giachetti, I., Aquino, D., Summers, P., … Gandini, C. A. M. (2021). White matter microstructure characterisation in temporal lobe epilepsy. bioRxiv. 10.1101/2021.05.08.442908.

[hbm25551-bib-0039] Sanz Leon, P., Knock, S. A., Woodman, M. M., Domide, L., Mersmann, J., Mcintosh, A. R., & Jirsa, V. K. (2013). The virtual brain: A simulator of primate brain network dynamics. Frontiers in Neuroinformatics, 7, 1–23. 23781198 10.3389/fninf.2013.00010 PMC3678125

[hbm25551-bib-0040] Schiavi, S., Ocampo‐Pineda, M., Barakovic, M., Petit, L., Descoteaux, M., Thiran, J. P., & Daducci, A. (2020). A new method for accurate in vivo mapping of human brain connections using microstructural and anatomical information. Science Advances, 6, 1–11. 10.1126/sciadv.aba8245 PMC7399649 32789176

[hbm25551-bib-0041] Schilling, K. G., Nath, V., Hansen, C., Parvathaneni, P., Blaber, J., Gao, Y., … Landman, B. A. (2019). Limits to anatomical accuracy of diffusion tractography using modern approaches. NeuroImage, 185, 1–11. 30317017 10.1016/j.neuroimage.2018.10.029 PMC6551229

[hbm25551-bib-0042] Schirner, M., Mcintosh, A. R., Jirsa, V. K., & Deco, G. (2018). Inferring multi‐scale neural mechanisms with brain network modelling. eLife, 7, 1–30. 10.7554/eLife.28927 PMC5802851 29308767

[hbm25551-bib-0043] Schmahmann, J. D., & Caplan, D. (2006). Cognition, emotion and the cerebellum. Brain, 129, 290–292. 16434422 10.1093/brain/awh729

[hbm25551-bib-0044] Schmahmann, J. D., & Pandya, D. N. (1995). Prefrontal cortex projections to the basilar pons in rhesus monkey: Implications for the cerebellar contribution to higher function. Neuroscience Letters, 199, 175–178. 8577391 10.1016/0304-3940(95)12056-a

[hbm25551-bib-0045] Simioni, A. C., Dagher, A., & Fellows, L. K. (2016). Compensatory striatal‐cerebellar connectivity in mild‐moderate Parkinson's disease. Neuroimage Clinical, 10, 54–62. 26702396 10.1016/j.nicl.2015.11.005 PMC4669533

[hbm25551-bib-0046] Smith, R. E., Tournier, J.‐D., Calamante, F., & Connelly, A. (2012). Anatomically‐constrained tractography: Improved diffusion MRI streamlines tractography through effective use of anatomical information. NeuroImage, 62, 1924–1938. 22705374 10.1016/j.neuroimage.2012.06.005

[hbm25551-bib-0047] Steele, C. J., Anwander, A., Bazin, P. L., Trampel, R., Schaefer, A., Turner, R., … Villringer, A. (2017). Human cerebellar sub‐millimeter diffusion imaging reveals the motor and non‐motor topography of the dentate nucleus. Cerebral Cortex, 27, 4537–4548. 27600851 10.1093/cercor/bhw258

[hbm25551-bib-0048] Streng, M. L., & Krook‐Magnuson, E. (2020). The cerebellum and epilepsy. Epilepsy & Behavior (In press). 10.1016/j.yebeh.2020.106909 PMC7415499 32035793

[hbm25551-bib-0049] Strick, P. L., Dum, R. P., & Fiez, J. a. (2009a). Cerebellum and nonmotor function. Annual Review of Neuroscience, 32, 413–434. 10.1146/annurev.neuro.31.060407.125606 19555291

[hbm25551-bib-0050] Tournier, J. D., Smith, R., Raffelt, D., Tabbara, R., Dhollander, T., Pietsch, M., … Connelly, A. (2019). MRtrix3: A fast, flexible and open software framework for medical image processing and visualisation. NeuroImage, 202, 116137. 31473352 10.1016/j.neuroimage.2019.116137

[hbm25551-bib-0051] Tournier, J.‐D., Calamante, F., & Connelly, A. (2007). Robust determination of the fibre orientation distribution in diffusion MRI: Non‐negativity constrained super‐resolved spherical deconvolution. NeuroImage, 35, 1459–1472. 17379540 10.1016/j.neuroimage.2007.02.016

[hbm25551-bib-0052] van Essen, D. C., Smith, S. M., Barch, D. M., Behrens, T. E. J., Yacoub, E., & Ugurbil, K. (2013). The WU‐Minn human connectome project: An overview. NeuroImage, 80, 62–79. 23684880 10.1016/j.neuroimage.2013.05.041 PMC3724347

[hbm25551-bib-0053] Voogd, J., & Ruigrok, T. J. H. (2012). Cerebellum and precerebellar nuclei & The human nervous system (3rd ed., pp. 471–545). San Diego, CA: Academic Press.

[hbm25551-bib-0054] Yu, H., Sternad, D., Corcos, D. M., & Vaillancourt, D. E. (2007). Role of hyperactive cerebellum and motor cortex in Parkinson's disease. NeuroImage, 35, 222–233. 17223579 10.1016/j.neuroimage.2006.11.047 PMC1853309

[hbm25551-bib-0055] Zhang, H. Y., Tang, H., Chen, W. X., Ji, G. J., Ye, J., Wang, N., … Guan, B. (2015). Mapping the functional connectivity of the substantia nigra, red nucleus and dentate nucleus: A network analysis hypothesis associated with the extrapyramidal system. Neuroscience Letters, 606, 36–41. 26342496 10.1016/j.neulet.2015.08.029

[hbm25551-bib-0056] Zimmermann, J., Perry, A., Breakspear, M., Schirner, M., Sachdev, P., Wen, W., … Solodkin, A. (2018). Differentiation of Alzheimer's disease based on local and global parameters in personalized virtual brain models. Neuroimage Clinical, 19, 240–251. 30035018 10.1016/j.nicl.2018.04.017 PMC6051478

